# P2X7 Receptor Upregulation in Huntington’s Disease Brains

**DOI:** 10.3389/fnmol.2020.567430

**Published:** 2020-10-06

**Authors:** Ivana Ollà, María Santos-Galindo, Ainara Elorza, José J. Lucas

**Affiliations:** ^1^Centro de Biología Molecular ‘Severo Ochoa’ (CBMSO) CSIC/UAM, Madrid, Spain; ^2^Networking Research Centre on Neurodegenerative Diseases (CIBERNED), Madrid, Spain

**Keywords:** P2X7, purines, ATP, Huntington’s disease, neuroinflammation, neurodegenerative disease

## Abstract

Huntington’s disease (HD) is a fatal degenerative disorder affecting the nervous system. It is characterized by motor, cognitive, and psychiatric dysfunctions, with a late onset and an autosomal dominant pattern of inheritance. HD-causing mutation consists in an expansion of repeated CAG triplets in the huntingtin gene (*HTT*), encoding for an expanded polyglutamine (polyQ) stretch in the huntingtin protein (htt). The mutation causes neuronal dysfunction and loss through multiple mechanisms, affecting both the nucleus and cytoplasm. P2X7 receptor (P2X7R) emerged as a major player in neuroinflammation, since ATP – its endogenous ligand – is massively released under this condition. Indeed, P2X7R stimulation in the central nervous system (CNS) is known to enhance the release of pro-inflammatory cytokines from microglia and of neurotransmitters from neuronal presynaptic terminals, as well as to promote apoptosis. Previous experiments performed with neurons expressing the mutant huntingtin and exploiting HD mouse models demonstrated a role of P2X7R in HD. On the basis of those results, here, we explore for the first time the status of P2X7R in HD patients’ brain. We report that in HD postmortem striatum, as earlier observed in HD mice, the protein levels of the full-length form of P2X7R, also named P2X7R-A, are upregulated. In addition, the exclusively human naturally occurring variant lacking the C-terminus region, P2X7R-B, is upregulated as well. As we show here, this augmented protein levels can be explained by elevated mRNA levels. Furthermore, in HD patients’ striatum, P2X7R shows not only an augmented total transcript level but also an alteration of its splicing. Remarkably, P2X7R introns 10 and 11 are more retained in HD patients when compared with controls. Taken together, our data confirm that P2X7R is altered in brains of HD subjects and strengthen the notion that P2X7R may represent a potential therapeutic target for HD.

## Introduction

Huntington’s disease (HD) is a neurodegenerative disorder characterized by motor dysfunction, cognitive decline, and psychiatric symptoms (McColgan and Tabrizi, 2018). The HD-causing mutation lies in the huntingtin (*HTT*) gene. In normal population, *HTT* exon 1 includes from 6 to 35 repeats of the CAG triplet, while HD patients have 40 or more repeats (MacDonald et al., 1993). This mutation has an autosomal dominant inheritance, is highly penetrant, and initiates the disease through various mechanisms (Duyao et al., 1993). On one hand, the expanded CAG repeats of the *HTT* mRNA are able to trap several RNA-binding proteins (Mykowska et al., 2011), thus likely provoking their loss of function. Interestingly, splicing factors and spliceosome components are among the sequestered proteins (Schilling et al., 2019). As a consequence, at least two pathogenic mis-splicing events, affecting *HTT* and *MAPT*, have been reported (Fernández-Nogales et al., 2014; Neueder et al., 2018). On the other hand, the expanded CAG triplets encode for an abnormally long polyglutamine (polyQ) trait in the N-terminus of the huntingtin protein (htt), which accounts for a toxic gain of function, as well (MacDonald et al., 1993). Both exon 1 and exon 1-like fragments contain the expanded polyQ trait and have been reported to trigger HD through toxic protein–protein interactions. Besides, htt fragments are prone to aggregate into microaggregates, fibrils, and inclusion bodies, which in turn may sequester other proteins, both in the nucleus and in the cytoplasm (Bates et al., 2015). All this leads to global neuronal impairment and death. Medium spiny neurons of the striatum are the most vulnerable cell type to mutant htt, which triggers striatal atrophy, the main hallmark in HD patients (McColgan and Tabrizi, 2018). Nevertheless, mutant htt is expressed throughout the whole brain, provoking degeneration in many other regions of the CNS.

P2X7 receptor (P2X7R) is a cation channel modulated by endogenous ATP. A single P2X7R subunit comprises a short intracellular N-terminus, two transmembrane motifs (TM1 and TM2) separated by an extracellular loop, and an intracellular C-terminus tail. P2X7R subunits coassemble in a trimeric chalice-like architecture provided of three ATP binding pockets (McCarthy et al., 2019; Rump et al., 2020). When ATP binds to P2X7R, the channel is open, allowing the transit of small cations, e.g., Na^+^, Ca^2+^, and K^+^ (Surprenant et al., 1996). However, when the ATP activation is prolonged or repeated, P2X7R acts as a non-selective pore, which allows transit of large-molecular-weight molecules (Surprenant et al., 1996). It has been shown that both the TM2 domain (Sun et al., 2013) and the C-terminus (Alloisio et al., 2010) play a pivotal role in the pore formation. P2X7R is expressed in many cell types, including neurons and brain glial cells (microglia, astrocytes, and Muller cells) (Armstrong et al., 2002; Hervás et al., 2003; Miras-Portugal et al., 2003). ATP is released as cotransmitter via synaptic vesicles, and thus, a transient and localized increase in extracellular ATP can follow neuronal activity. Accordingly, a function of P2X7R in neuron–neuron communication and plasticity has been established (Sperlágh et al., 2006). However, wider ATP release is observed in response to adverse events, including ischemia, hypoxia, mechanical stimuli, bacteria, or toxin exposure (Csölle and Sperlágh, 2010). This kind of high levels of extracellular ATP leads to P2X7R-mediated neuron–glia crosstalk and glial activation, which in turn causes P2X7R upregulation. In general, P2X7R stimulation leads to a Ca^2+^ influx, which has different consequences depending on the cell type where it occurs. P2X7R provokes glutamate release from presynaptic nerve terminals and from astrocytes, responsible for an excitotoxic effect (Sperlágh et al., 2002), while astrocytes and microglia are accountable for IL-1β, IL-6, and TNF-α release, triggering neuroinflammation (Solle et al., 2001). ROS production, combined with BDNF downregulation, is a further mechanism by which neuronal damage and reactive gliosis are achieved via P2X7R (Ficker et al., 2014). Under prolonged stimulation, P2X7R pore opens, prompting plasma membrane blebbing and lastly cell death (Sorge et al., 2012). All this makes P2X7R a key player in neuroinflammation.

Notably, different P2X7R transcript variants deriving from alternative splicing exist in both human and mouse. In particular, 10 isoforms (from *A* to *J*) have been described for the human receptor (Cheewatrakoolpong et al., 2005). *P2* × *7R-A* comprises 13 exons and corresponds to the canonical transcript (Buell et al., 1998). *P2* × *7R-B* differs from it for the retention of the 84-nucleotide-long intronic region between exon 10 and exon 11, while variants *P2* × *7R-C/D/E/F/J* lack either exon 4, 5, 7, 8, or 7 and 8 together. *P2* × *7R-G* and *P2* × *7R-H* have an extra exon named N3 between exon 2 and exon 3. *P2* × *7R-I* lacks both exon 2 and N3. Transcripts *E*, *G*, and *I* also present the intron 10–11 retention. Of such transcripts, four have been extensively studied since they originate proteins. Therefore, not a unique P2X7R exists. Rather, four P2X7Rs have been described based on alternative splicing: P2X7R-A, P2X7R-B, P2X7R-H, and P2X7R-J (Cheewatrakoolpong et al., 2005; Feng et al., 2006).

*P2* × *7R-A* encodes the well-characterized full-length P2X7R-A. It includes 595 aa constituting the N-terminus, TM1 and TM2 separated by an extracellular loop, and the intracellular C-terminus of the protein. The N-terminus can form intracellular complexes with many substrates including heat shock proteins, β2-integrin, α-actin, and several protein kinases and phosphatases (Kim et al., 2001). The extracellular loop owns the ligand-binding sites and a number of N-glycosylation sites (Wang et al., 2005). The C-terminus of P2X7R-A, due to multiple protein–protein and protein–lipid interaction motifs (Denlinger et al., 2001), contributes to its communication with cytoskeletal and intracellular proteins (Kim et al., 2001) and is required for the formation of a pore, hence eliciting many functions of the receptor. *P2* × *7R-B* is the transcript for P2X7R-B and lacks the C-terminus as a consequence of the premature stop codon introduced by the intron 10–11 retention. Accordingly, this protein comprises 364 aa, where the last 18 aa are different from those of P2X7R-A. Interestingly, *P2* × *7R-B* seems to be the predominant P2X7R transcript in multiple human tissues, including the brain (Cheewatrakoolpong et al., 2005; Adinolfi et al., 2010). Experiments in HEK293 cells expressing P2X7R-B demonstrate its ability to form homotrimers and maintain all the ATP-stimulated channel functions, despite being unable to form a non-selective pore and trigger apoptosis. Thus, P2X7R-B is free of the cytotoxic activity linked to the C-terminal tail and is generally considered a less “dangerous” form of P2X7R. However, when coexpressed, P2X7R-A and P2X7R-B can heterotrimerize efficiently. In this case, P2X7R-B potentiates P2X7R-A functions, including the formation of a pore and proapoptotic activity. Therefore, cells could modulate ATP responses by P2X7R-A and P2X7R-B expression ratio and combination in trimers (Adinolfi et al., 2010). A pathophysiological role of P2X7R-B has been described in multiple conditions, including bone cancer (Giuliani et al., 2014) and bone differentiation (Carluccio et al., 2019). It has also been described in neural progenitors (Glaser et al., 2014), neuroblastoma cells (Ulrich et al., 2018), and glioblastoma cells (Ziberi et al., 2019). P2X7R-H contains 505 aa and is also known as P2X7R-ΔTM1, since the TM1 is absent. Indeed, *P2* × *7R-H* contains the N3 exon, which creates a new start codon responsible for the absence of the first part of the protein. However, when transfected in HEK293 cells, P2X7R-H is an inactive receptor (Cheewatrakoolpong et al., 2005). P2X7R-J includes only 258 aa and lacks the C-terminus, the TM2, and part of the extracellular loop. Still, P2X7R-J can form heterotrimers with P2X7R-A. It emerged to act as a dominant negative, since it antagonizes the function of P2X7R-A in cervical cancer cells (Feng et al., 2006). To date, the implication of such variety around P2X7Rs expression in both physiological and pathological backgrounds has never been explored in the nervous system.

Cell injury is a common feature of neuroinflammatory and neurodegenerative disorders, including traumatic brain injury, stroke, epilepsy, neuropathic pain, amyotrophic lateral sclerosis, multiple sclerosis, Alzheimer’s disease, Parkinson’s disease, and HD. When damaged, cells release a great amount of ATP, which acts on P2X7R. Therefore, P2X7R has been proposed as a pivotal player in all these conditions and a potential common target for their treatment (Díaz-Hernández et al., 2009; Kim et al., 2009; Sanz et al., 2009; Amadio et al., 2011; Lämmer et al., 2011; Kimbler et al., 2012; North and Jarvis, 2013; Carmo et al., 2014). Besides, and regarding HD, P2X7R upregulation has been described in the striatum of R6/1 and Tet/HD94 mouse models of HD (Díaz-Hernández et al., 2009). Moreover, cultures of primary neurons from such mice showed that P2X7R exhibits an altered permeability to calcium, suggesting a different functional state of the receptor and higher vulnerability to P2X7R-mediated apoptosis (Díaz-Hernández et al., 2009), thus indicating a direct link between P2X7R and HD. Remarkably, the report of a lower rate of neuronal apoptosis and motor impairment recovery in HD mice following the administration of Brilliant Blue-G (BBG), a P2X7R antagonist, further suggested a contribution of P2X7R to this pathology (Díaz-Hernández et al., 2009), pinpointing P2X7R blockade as a possible therapeutic approach for HD. As, to date, P2X7R has not been explored in brains of HD subjects, here, we aim to further validate the possible role of P2X7 in HD pathogenesis.

## Materials and Methods

### Human Brain Tissue Samples

Striatal tissues, including the caudate and putamen, from control and HD individuals were provided by Banco de Tejidos Fundación Cien (BT-CIEN, Madrid, Spain; CTRL *n* = 5, HD *n* = 7), the Netherlands Brain Bank (Amsterdam, The Netherlands; CTRL *n* = 4, HD *n* = 5), and Banc de Teixits Neurològics (Barcelona, Spain; CTRL *n* = 6, HD *n* = 6). Controls and HD subjects are matched by age (CTRL = 56 ± 3.8; HD = 63 ± 3.5) and sex (of the total, CTRL: *M* = 70%, *F* = 30%; in HD: *M* = 60%, *F* = 40%). Postmortem interval (PMI) is lower than 24 h in all cases. HD subjects’ mean of CAG repetitions is 45.7 ± 1.4. Written informed consent for brain removal after death for diagnostic and research purpose was obtained from brain donors and/or next of kin.

### Antibodies

In order to explore P2X7Rs at the protein level in brain samples, different antibodies have been employed:

**Table:** 

**Host**	**Brand**	**Reference**	**Directed to**	**Epitope location**	**Epitope sequence**	**Detects**
Rabbit	Alomone Lab	APR- 004	C-terminal	rat epitope 576–595	KIRKEFPKTQ GQYSGFKYPY	P2X7R-A
						P2X7R-H
Goat	NovusBio	NBP1- 37775	N-terminus	human epitope 13–26	YETNKV TRIQSMNY	P2X7R-B
						P2X7R-J
Rabbit	Alomone Lab	APR- 008	extracellular	mouse epitope 136–152	KKGWMDP QSKGIQTGRC	all P2X7Rs

### Western Blot Analysis

Different cohorts of samples from human brains were stored at −80°C and ground with a mortar in a frozen environment with liquid nitrogen to prevent thawing of the samples, resulting in tissue powder. Protein extracts were prepared by homogenizing tissue powder in ice-cold extraction buffer (20 mM HEPES pH 7.4, 100 mM NaCl, 20 mM NaF, 1% Triton X-100, 1 mM sodium orthovanadate, 1 μM okadaic acid, 5 mM sodium pyrophosphate, 30 mM β-glycerophosphate, 5 mM EDTA, protease inhibitors (Complete, Roche, cat. no. 11697498001). Homogenates were centrifuged at 15,000 rpm for 15 min at 4°C. The resulting supernatant was collected, and protein content determined by Quick Start Bradford Protein Assay (Bio-Rad, 500-0203). Ten micrograms of total protein was electrophoresed on 10% SDS-polyacrylamide gel, transferred to a nitrocellulose blotting membrane (Amersham Protran 0.45 μm, GE Healthcare Life Sciences, 10600002), and blocked in TBS-T (150 mM NaCl, 20 mM Tris–HCl, pH 7.5, 0.1% Tween 20) supplemented with 5% non-fat dry milk. Membranes were incubated overnight at 4°C with different anti-P2X7R antibodies directed to C-terminus (rabbit, 1:1,000, Alomone Lab, APR-004), N-terminus (goat, 1:500, NovusBio, NBP1-37775), or extracellular domain (rabbit, 1:200, Alomone Lab, APR-008) in TBS-T supplemented with 5% non-fat dry milk, washed with TBS-T, and next incubated with HRP-conjugated anti-rabbit IgG (1:2,000, DAKO, P0448) or anti-goat IgG (1:5,000, Bethyl, A50-101P) and developed using the ECL detection kit (PerkinElmer, NEL105001EA). As a loading control, β-actin (1:50,000, Sigma, A2066), α-tubulin (1:20,000, Sigma, T9026), and vinculin (1:20,000, Abcam, ab129002) were used. Densitometric analysis was carried out by using a densitometer (Bio-Rad GS800). Quantification was performed by using Image Lab 5.2 software (Bio-Rad). In all cases, the average intensity value of the pixels in a background-selected region was calculated and was subtracted from each pixel in the samples. The densitometry values obtained in the linear range of detection with the antibody was normalized with respect to loading controls to correct for any deviation in loaded amounts of protein.

### Immunohistochemistry

Formalin-fixed (4%, 24 h), paraffin-embedded tissues from the striatum were used (CTRL *n* = 3, HD *n* = 3). Sections (5 μm thick) were mounted on SuperFrost Plus tissue slides (Menzel Gläser) and deparaffinized. Brain sections were immersed in 0.3% H_2_O_2_ in methanol for 45 min to quench endogenous peroxidase activity. Sections were then immersed for 1 h in blocking solution (PBS containing 0.5% fetal bovine serum, 0.3% Triton X-100, and 1% BSA) and incubated overnight at 4°C with C-terminal-directed anti-P2X7R (rabbit, 1:1,000, Alomone Lab, APR-004) or N-terminal-directed anti-P2X7R (goat, 1:500, NovusBio, NBP1-37775), diluted in blocking solution. After washing, brain sections were incubated first with biotinylated anti-rabbit or anti-goat secondary antibody and then with avidin–biotin complex using the Elite VECTASTAIN kit (Vector Laboratories, PK-6101 and PK-6105). Chromogen reactions were performed with diaminobenzidine (SIGMAFAST DAB, Sigma, D4293) for 10 min. Sections where first dehydrated and then mounted with DePeX (SERVA). Images were captured using an Olympus BX41 microscope with an Olympus camera DP-70 (Olympus Denmark A/S).

### RNA Extraction and cDNA Synthesis

Total tissue RNA was extracted from the striatum of CTRL and HD patients using the Maxwell^®^ 16 LEV simplyRNA Tissue Kit (Promega, AS1280). Quantification and quality of RNA were done on a NanoDrop ND-1000 spectrophotometer and NanoDrop 1000 v.3.7.1 (Thermo Scientific). Retrotranscription (RT) reactions were performed using the iScript cDNA synthesis kit (Bio-Rad, PN170-8891) following manufacturer’s instructions. Briefly, 1 μg of total RNA from each sample and 4 × Master Mix (which includes all necessary reagents, a mixture of random primers, and oligo-dT for priming) were brought to a final volume of 40 μl with DNase/RNase-free distilled water (Gibco, PN10977). Thermal conditions consisted of 5 min at 25°C, 30 min at 46°C, and 5 min at 95°C.

### P2X7R Quantitative and Semi-Quantitative PCR

Quantitative RT-PCR was performed using gene-specific primers and TaqMan MGB probes for human P2X7R (forward, 5′-GTGAACCAGCAGCTACTAGGGAG-3′; reverse, 5′-TGAAGTCCATCGCAGGTCTTG-3′), β-actin, and GAPDH. Fast thermal cycling was performed using a StepOnePlus Real-Time PCR System (Applied Biosystems) as follows: denaturation, one cycle of 95°C for 20 s, followed by 40 cycles each of 95°C for 1 s and 60°C for 20 s. The results were normalized as indicated by parallel amplification of the endogenous controls β-actin and GAPDH. Semi-quantitative RT-PCR was performed by designing specific primers in P2X7R exon 10 and exon 11 (forward, 5′-CATCGGCTCAACCCTCTCCTA-3′; reverse, 5′-TTTGGCTCCACAATGGACTCG-3′) to amplify the intron 10–11 retaining isoforms in human brain cDNA. PCR amplification protocol used 5 min 94°C + 30 cycles (30 s at 94°C + 30 s at 58°C + 2 min at 72°C) + 7 min at 72°C. The PCR product was resolved on 1.5% agarose/GelGreen (Biotium, 41004) gels run at 120 V for 1.5 h. Images were taken in a UviDoc transilluminator (UviTec) and then scanned with densitometer (Bio-Rad, GS-900) and quantified with Image Lab 5.2 (Bio-Rad).

### P2X7R Transcript Level and Splicing Alteration in HD by RNA-Seq

P2X7R expression levels in HD and CTRL postmortem striatum samples were evaluated from our RNA-seq data (CTRL = 3 vs. HD = 3) (Elorza et al., 2020). For total mRNA transcript levels, reads were aligned against the *Homo sapiens* genome (GRCh38.p2 version) using the TopHat2 aligner (Trapnell et al., 2009), and differentially expressed genes were obtained with the Cuffdiff software (Trapnell et al., 2013). For the splicing analysis, percent spliced in (PSI) values were obtained by running vast-tools (Vertebrate Alternative Splicing and Transcription Tools) (Irimia et al., 2014). Alternatively, TopHat-aligned reads were run with the rMATS software for the detection of altered spliced events (Shen et al., 2014).

### Statistics

All experiments were repeated at least three times, and the results are presented as the mean ± sem. Statistical analysis was performed with SPSS 21.0 (SPSS Statistic IBM). Where indicated, Student’s *t*-test was applied, and false discovery rate (FDR) was calculated in the case of RNA-seq data.

## Results

### P2X7R Protein Level Is Increased in the Striatum of HD Patients

**FIGURE 1:**
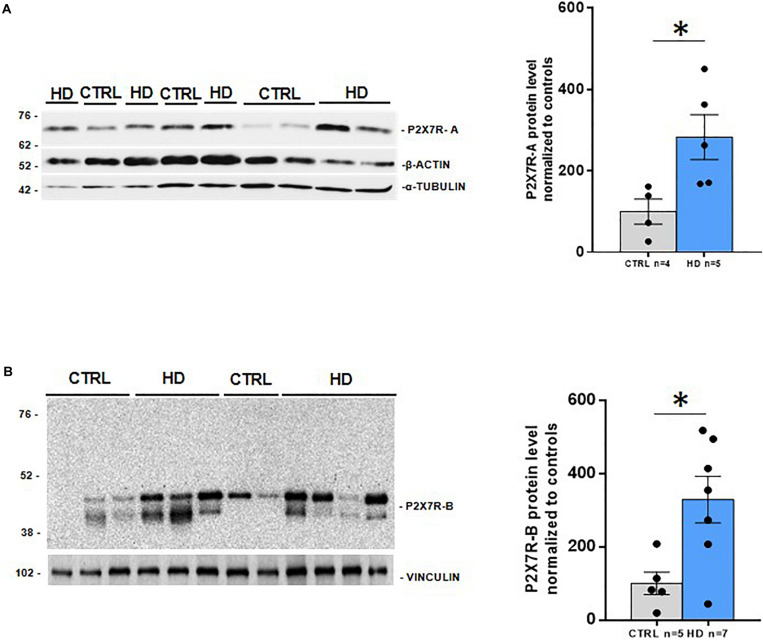
Increased P2X7R protein levels in striatal post-mortem samples from HD patients. Western blot analysis of **(A)** P2X7R-A (CTRL *n* = 4, HD *n* = 5) with a C-terminus oriented antibody, and **(B)** P2X7R-B (CTRL *n* = 5, HD *n* = 7) with a N-terminus directed antibody, with their loading controls (below) and the corresponding quantifications (right). Graphs show mean ± SEM. Dots represent individual values. Student’s *t*-test, *p < 0.05.

To further explore the potential role of P2X7R in HD pathogenesis, we first analyzed by western blot the protein level of this receptor in the striatum of HD subjects, the most affected brain region in patients. As previously mentioned, human P2X7R mRNA can undergo differential splicing, thus originating multiple P2X7R variants. Of these, four are protein encoding and generate the P2X7R-A, P2X7R-B, P2X7R-H, and P2X7R-J isoforms, with expected molecular weights of 68.6, 41.8, 58.2, and 29.3 kDa, respectively. In order to detect P2X7R-A and P2X7R-H, which are provided with in the canonical C-terminus, we used a C-terminus-directed antibody. With this antibody, we observed a unique band of about 70 kDa, most likely corresponding to P2X7R-A, which was increased (2.8-fold, *p* = 0.03) in brain striatal extracts of HD patients (Figure 1A). This P2X7R upregulation in HD specimens is in accordance with the one previously obtained in the same brain region in two mouse models of HD (Díaz-Hernández et al., 2009). P2X7R-H, which indeed presents the same C-terminus sequence of P2X7R-A, has not been detected with this antibody, even after a prolonged exposure, neither in normal nor in pathological conditions. Regarding the C-terminus truncated isoforms, P2X7R-B and P2X7R-J, we opted for an N-terminus-directed antibody, which should also recognize the full-length form. First, after short exposure, we observed a doublet at 42–45 kDa, possibly corresponding to P2X7R-B. Such doublet results strongly increased (3.3-fold, *p* = 0.01) in the striatum of HD patients (Figure 1B). Second, following long exposure, a band can be observed in the range of 30–38 kDa, possibly corresponding to P2X7R-J (Supplementary Figure S1). In HD, such band shows a non-statistically significant increase due to the great dispersion of data within the HD group (*p* = 0.2). This antibody also detects a doublet at approximately 60 kDa. Such a molecular weight could correspond to a homodimerization of P2X7R-J; however, no change occurs between control and HD specimens (*p* = 0.5). Finally, a shade at approximately 70 kDa, possibly corresponding to P2X7R-A, can be detected. It seems, therefore, that this antibody is somehow less efficient than the C-terminus-directed one in detecting the full-length protein (Supplementary Figure S1). Since the extracellular loop represents a shared feature of all P2X7R isoforms, an extracellular loop-directed antibody has been used for the simultaneous detection of P2X7R-A and P2X7R-B (Carluccio et al., 2019; Ziberi et al., 2019). Unfortunately, this antibody was not sensitive enough to detect P2X7R in human postmortem brain samples (data not shown). Taken together, these results indicate that P2X7R is upregulated at the protein level in HD, with an augmented level of both full-length (P2X7R-A) and C-terminus lacking (P2X7R-B) forms of the receptor.

### P2X7R Immunoreactivity Is Augmented in HD Brains

**FIGURE 2:**
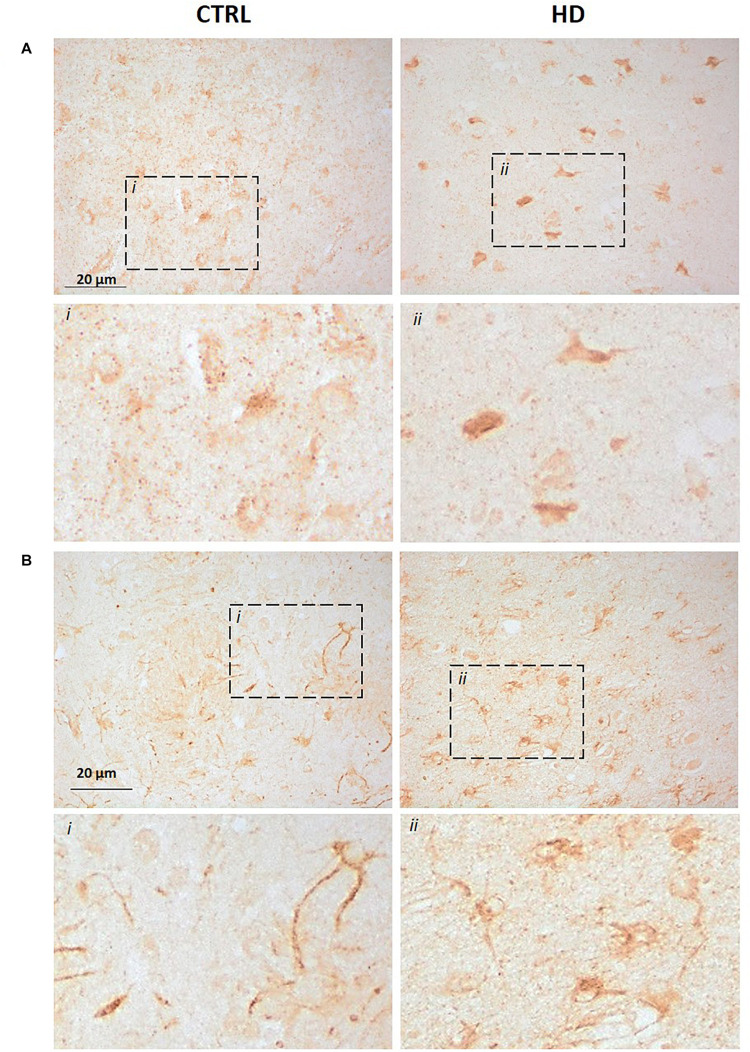
Increased P2X7R-associated immunoreactivity in striatal sections from HD patients (CTRL *n* = 3, HD *n* = 3). Immunohistochemistry assay was carried out with **(A)** the C-terminus directed antibody or **(B)** the N-terminus directed antibody. In both cases, an augmented signal was observed in HD sections when compared to controls. Dash line boxes show the zoomed areas (i;ii) in both cases.

We further investigated P2X7R status in HD brains through immunohistochemistry, with the same two antibodies that were used for the western blot analysis. Detection of P2X7R immunoreactivity with the C-terminal antibody in the striatum of control subjects shows a weak punctate and cytoplasmic pattern mainly in neurons and neuropil; in striatal sections from HD patients, we observe a more diffuse and intense reactivity, which in neurons appears to extend to their processes, and a higher number of clearly immunoreactive cells was seen (Figure 2A). Since no P2X7R-H-associated band has been detected by western blot in this brain region, we can speculate that the signal detected here essentially corresponds to P2X7R-A.

Regarding the N-terminal antibody, the pattern in the striatum of controls was also cytoplasmic and punctate mainly in neurons, but neuronal processes also get clearly stained. This pattern seems to be maintained in HD striatal sections, but with more abundant immunoreactive somata (Figure 2B). Since by western blot this antibody identified mainly P2X7-B, while other bands emerged only after a prolonged exposure, we can speculate that such signal could essentially be related to P2X7R-B. This result, together with the increased protein level detected by western blot, highlights an alteration of P2X7R-A and P2X7R-B in HD brains.

### P2X7R Transcript Alteration in HD Brains

**FIGURE 3:**
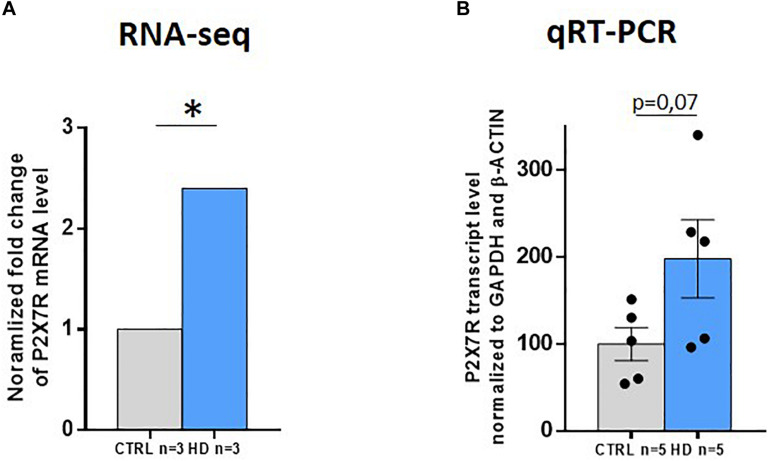
P2X7R total transcript levels strongly increase in HD striatum. **(A)** P2X7R transcript level analyzed by RNA-seq (CTRL *n* = 3, HD *n* = 3). Fold-change with respect to controls. q = adjusted *p*-value. *q < 0.05. **(B)** P2X7R total transcript levels by qRT-PCR (CTRL *n* = 6, HD *n* = 6). GAPDH and β-actin were used as housekeeping genes for normalization. Student’s *t*-test. Graphs show mean ± SEM. Dots represent individual values.

Since P2X7R protein levels are augmented in HD striatum, we investigated whether this increase is associated with a higher amount of total P2X7R transcript in this brain area. To achieve such aim, we analyzed an RNA-seq study of the striatum of HD patients and controls that we have recently performed (Elorza et al., 2020). We found a strong increase in total P2X7R transcript level (2.4-fold, *q* = 0.005) in HD subjects (Figure 3A). We further analyzed P2X7R mRNA level in the striatum by quantitative-PCR (Q-PCR) in an independent cohort of patients. Coherently, we detected a tendency of having a higher amount of P2X7R transcripts (2.0-fold, *p* = 0.07) in this region when comparing HD subjects with controls (Figure 3B). Taken together, these results suggest that the striatal increase in P2X7R protein levels detected in HD is likely associated with augmented P2X7R total transcript levels in the same brain region. This is consistent with the previously reported trend of an increase in P2X7R mRNA in the brain of R6/1 and Tet/HD94 mouse models of HD (Díaz-Hernández et al., 2009).

**FIGURE 4:**
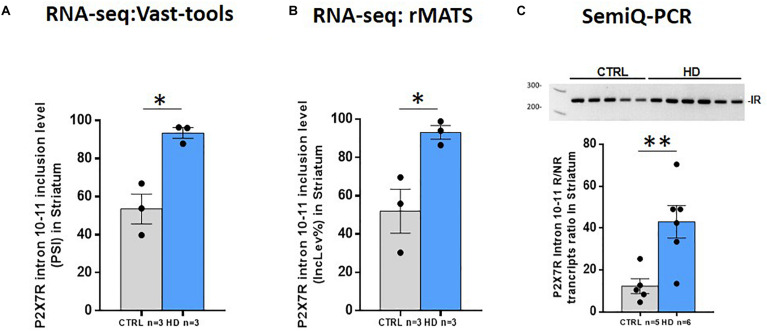
P2X7R altered intron retention in HD striatum. **(A)** Percentage of spliced in (PSI) values for P2X7R intron 10–11 retention in control and HD according to Vast-tools analysis (ΔPSI = 39%). Student’s *t*-test. *p < 0.05. **(B)** P2X7R intron 10–11 inclusion level percentage (IncLev%) for control and HD according to rMATS analysis (IncLevDiff% = 41%). *FDR < 0.05. **(C)** Semi-qRT-PCR directed to P2X7R intron 10–11, represented as the ratio between intron retaining/non-retaining transcripts, confirms increased retention in independent HD cases (CTRL *n* = 5, HD *n* = 6). Student’s *t*-test. **p < 0.01. Graphs show mean ± sem. Dots represent individual values.

On one hand, previous data have shown the existence of naturally occurring isoforms of P2X7R generated by differential splicing, which could play a role in physiological and/or pathological conditions (Cheewatrakoolpong et al., 2005; Feng et al., 2006; Adinolfi et al., 2010). On the other hand, splicing alterations have been reported to contribute to HD pathogenesis (Fernández-Nogales et al., 2014; Neueder et al., 2018). Thus, we wondered whether P2X7R suffers from splicing alterations in HD, as well. We therefore analyzed the RNA-seq study performed in the striatum with two different bioinformatic tools. By using vast-tools, we found that the inclusion level of the 84-nucleotide-long intronic region located between exon 10 and exon 11 is increased by 39% in the HD striatum (*p* = 0.007, Figure 4A). We also found that exon 4 inclusion was decreased about 12% in patients (*p* = 0.01, Supplementary Figure S2A). When we performed the equivalent analysis by using rMATS, we confirmed that intron 10–11 retention is increased by 41% (FDR = 0.002) (Figure 4B) and that exon 4 inclusion is about 8% lower (FDR = 0.04) in HD patients (Supplementary Figure S2B).

While no function has been annotated regarding exon 4, there is literature about intron 10–11 retention. As mentioned, it is involved in the production of the P2X7R-B protein (Cheewatrakoolpong et al., 2005), which has been described to play a role *in vitro* (Adinolfi et al., 2010). Therefore, we validated in an independent set of samples the increased retention of intron 10–11 by semi-quantitative RT-PCR with primers hybridizing in the flanking exons (10 and 11). This confirmed that, indeed, intron 10–11 are more retained in HD postmortem brains, since the proportion of transcripts including this region with respect to the non-intron-containing ones is more abundant in our HD sample set (3.5-fold, *p* = 0.008) (Figure 4C). Taken together, these results demonstrate that, in the striatum of HD subjects, P2X7R undergoes mis-splicing with slightly decreased inclusion of exon 4 and markedly increased retention of intron 10–11, the latter explaining the increase in the P2X7R-B isoform observed by western blot.

## Discussion

Here, we perform, to our knowledge, the first analysis of P2X7R status in brains of HD subjects. By analyzing P2X7R proteins through western blot and immunohistochemistry and P2X7R transcript isoforms through RNA-seq and subsequent validation by RT-PCR, here, we report a clear increase in the total levels of P2X7R. Both the full-length isoform P2X7R-A and the lower-molecular-weight isoform P2X7R-B show increased levels that can be explained by the changes observed at the transcript level, the latter affecting both the total transcript levels and the increased retention of intron 10–11 which originate the P2X7R-B isoform.

To date, no disease-modifying treatment is available for HD subjects, who only receive palliative care. Although a number of clinical trials have been completed during the past years, no compelling results emerged. Thus, the research of new therapeutic approaches, possibly acting on the multiple features of the disease, remains open. Previous data demonstrated that P2X7R participates in the modulation of neurotransmitter release (Deuchars et al., 2001) and also in microglial (Ferrari et al., 1997) and astroglial activation (Verderio and Matteoli, 2001), thus highlighting a role of this receptor in neuroinflammation. Since neuroinflammation is a shared feature of many CNS diseases, including HD, P2X7R has been proposed to be a potential therapeutic target for their treatment. More compelling evidence of P2X7R as a likely therapeutic target specifically for HD arose from experiments performed in various HD cellular and mouse models, where the receptor was found to be upregulated and functionally altered (Díaz-Hernández et al., 2009), thus suggesting a role of P2X7R in the pathophysiology of HD. Interestingly, antagonizing P2X7R through the administration of BBG provokes less body weight loss and improves motor coordination in HD mice (Díaz-Hernández et al., 2009). Those results strongly supported the hypothesis of P2X7R as a target for the treatment of HD. However, evidence of P2X7R being in fact altered in the brains of HD subjects was missing. In order to address the status of P2X7R in HD brains, here, we focused on the striatum, the most affected brain region in the disease.

In the present study, we investigated the protein level in control and HD striatal samples first by western blot with an antibody raised against a C-terminal epitope. This antibody is expected to recognize the full-length P2X7R-A isoform. In good agreement with the expected weight of 68.6 kDa, we detected a band at around 70 kDa, thus fitting P2X7R-A. Interestingly, experiments performed on a cervix cancer cell line with the same antibody showed that P2X7R-A can reach the weight of 85 kDa (Wang et al., 2005) due to the N-glycosylation at five different sites (Asn-187, Asn-202, Asn-213, Asn-241, and Asn-284) (Surprenant et al., 1996). Since a unique band was identified in our study, it seems that the N-glycosylated P2X7R is not detectable in striatal samples. Moreover, although the antibody could potentially detect P2X7R-H as well, we did not observe any signal at its expected molecular weight. We therefore believe that the P2X7R-H isoform is not expressed or that it is expressed at a very low amount in the human striatum. In summary, the western blot experiments with the C-terminal antibody allowed us to conclude that P2X7R-A is expressed in the human striatum and is upregulated in HD patients (Figure 1A). This result is in accordance with the previous one obtained in R6/1 mice in the same brain region (Díaz-Hernández et al., 2009). We have also explored at protein level the P2X7R isoforms lacking the C-terminus, which are generated following differential splicing: P2X7R-B and P2X7R-J (Cheewatrakoolpong et al., 2005). We wondered whether these variants were expressed in the striatum and whether changes occur in the pathological context of HD. To address these questions, we used an antibody that binds an N-terminal sequence. We observed a doublet at 42/45 kDa possibly corresponding to P2X7R-B, which is upregulated in HD (Figure 1B). Regarding P2X7R-J, we observed a band between 30 and 38 kDa, which could correspond to such form, since the predicted molecular weight based on its sequence (UniProtKB–Q15G98) is 29.3 kDa. There is a non-significant tendency to an increase in HD regarding such band (*p* = 0.2) (Supplementary Figure S1). It must be considered that P2X7R-J has been discovered in cervical cancer cells (Feng et al., 2006). There, the group describes a 42/45 kDa doublet as P2X7R-J, as this isoform maintains four out of the five N-glycosylation sites of the full-length form that, after posttranslational modification, would increase its weight till 41.8 kDa. Since we did not obtain the 85-kDa band equivalent to the N-glycosylated form of P2X7R-A in our samples, we believe that the forms we are observing are the unglycosylated ones. In such condition and taking into consideration that the molecular weights of 42/45 kDa are closer to the one P2X7R-B is expected to show, 41.8 kDa, we consider that such doublet could correspond to P2X7R-B rather than to P2X7R-J.

The immunohistochemical analysis of P2X7R in striatal sections shows increased staining mainly in neurons, opposite to Alzheimer’s disease tissue and mouse models which show increased P2X7R staining in glial cells (Martin et al., 2019), possibly indicating a major role of increased P2X7R in altered neurotransmission rather than in neuroinflammation in HD. The immunohistochemical analysis has been carried out by exploiting the same antibodies used for the western blot analysis. Since the C-terminus-directed antibody substantially recognizes P2X7R-A while the N-terminus-directed one recognizes mostly the P2X7R-B-associated bands, we can assume that these are the proteins accountable for the observed immunoreactivities. However, we cannot exclude that the signal observed in this brain sections could be associated with other P2X7Rs not clearly detectable by western blot. Interestingly, the fact that the N-terminal antibody stains neuronal processes that are not detected with the C-terminus-directed antibody in control tissue suggests that P2X7R-A and P2X7R-B isoforms may have different spatial distributions in physiological conditions. Regardless of this, the increased number of somata with clear immunostaining detected with both antibodies is in accordance with the increased protein levels detected by western blot in HD. To date, we do not know the meaning of the increased protein level of both P2X7R-A and P2X7R-B in the neurons of HD patients. In this regard, it has been reported that P2X7R-A and P2X7R-B play a role in undifferentiated and neural-differentiated embryonic stem cells (ESC) (Glaser et al., 2014). The authors there demonstrated that while before differentiation both isoforms are well expressed by ESC, under neural differentiation, the ratio between P2X7R-A and P2X7R-B favors the full-length isoform (Glaser et al., 2014). They speculate that the good expression of P2X7R-B in the undifferentiated condition eases cell proliferation and differentiation, avoiding cellular death (Glaser et al., 2014). Indeed, P2X7R-B trophic activity had been previously described in HEK293 cells as well as in a human osteosarcoma cell line (Adinolfi et al., 2010; Giuliani et al., 2014). However, neurons in the striatum are post-mitotic cells, which do not enter cell cycle or differentiate. On one hand, it is possible to speculate that P2X7R-A and P2X7R-B increase in HD could represent a consequence of the inflammatory mechanisms related to HD. On the other hand, it is possible that such increase, and especially of P2X7R-B, could be an adaptive mechanism to favor neuronal survival in HD. Further experiments are required to establish P2X7R-associated pathophysiological mechanisms in neurons.

At the mRNA level, we observed increased total P2X7R mRNA levels in the HD striatum, which in turn may explain the increase in total protein levels, but we do not know the precise underlying mechanisms. These might take place at the transcriptional level, as there are multiple transcription factors altered in HD and global transcriptomic alteration is well-documented (Valor, 2015). Another possibility is that it is due to a posttranscriptional mechanism, for instance, involving microRNAs, given their ability to control stability and translation of their target transcripts. In this regard, it is known that the P2X7R transcript can be regulated by microRNA-22 (mir-22) (Jimenez-Mateos et al., 2015). More precisely, experiments performed in a mouse model of status epilepticus demonstrated that the P2X7R transcript is a target of mir-22, which normally inhibits its translation (Jimenez-Mateos et al., 2015). On the other hand, it has been reported that many microRNAs are downregulated in HD mouse models, including mir-22 (Lee et al., 2011). Indeed, the overexpression of mir-22 inhibited neurodegeneration in rat primary striatal cultures exposed to a mutated human huntingtin fragment (Htt171-82Q) (Jovicic et al., 2013). Thus, P2X7R increase in HD could be, at least in part, explained via mir-22 diminution in this pathology, but further investigation regarding the status of mir-22 in samples from HD patients would be necessary to ascertain this. Finally, and specifically regarding the increase in P2X7R-B isoform at both mRNA and protein levels, it can be explained by the increased retention of intron 10–11 in the P2X7R transcript in HD reported here. The likely underlying mechanism could be the already-reported alteration of many splicing factors in HD brain tissue and cell and mouse models such as MBLN1 (Mykowska et al., 2011) or SRSF6 (Fernández-Nogales et al., 2014; Cabrera and Lucas, 2017). Thus, we here provide for the first time a connection between HD dysregulated splicing and P2X7R-mediated mechanisms in HD. Nevertheless, further research is needed to identify the specific splicing factors binding near or at P2X7R intron 10–11.

In conclusion, here, we report an upregulation of P2X7R in HD brains that, together with the analogous increase in HD mouse models and the preclinical studies – also in the mouse models – showing efficacy of P2X7R manipulation, further provides evidence of a role of P2X7R in this pathology and strengthens its potential as a drug target.

## Data Availability Statement

The raw data supporting the conclusions of this article will be made available by the authors, without undue reservation.

## Ethics Statement

Ethical review and approval was not required for the study on human participants in accordance with the local legislation and institutional requirements. The patients/participants provided their written informed consent to participate in this study.

## Author Contributions

JL directed the study, designed experiments, performed the manuscript revision, and read and approved the submitted version. AE and IO performed the RNA sequencing analysis. IO performed q-RT-PCR and semi-q-RT-PCR analyses. MS-G carried out western blotting and the immunostaining. IO analyzed data and wrote the draft of the manuscript. All authors contributed to the article and approved the submitted version.

## Conflict of Interest

The authors declare that the research was conducted in the absence of any commercial or financial relationships that could be construed as a potential conflict of interest.

## Supplementary Material

FIGURE S1 P2X7R protein levels detected with an N-terminus directed antibody in control and HD striatum. Blot image (left) is able to highlight multiple bands after prolonged exposure. The band at around 30 kDa could correspond to P2X7R-J. As shown by its quantification (right), such band does not significantly vary between HD and controls. Doublets can be observed at around 60 kDa, which could correspond to P2X7R-J dimers. However, no significant change occurs in HD samples. Only a light shade is observed in the range of P2X7R-A. Graphs show mean ± SEM. Dots represent individual values. Student’s *t*-test, ns: non-significant.

FIGURE S2 P2X7R exon 4 inclusion is decreased in HD. RNA-seq analysis of control and HD striatum by **(A)** Vast-tools result (Student’s *t*-test, ^∗^*p* < 0.05) was confirmed by **(B)** rMATS (^∗^FDR < 0.05). Graphs show mean ± SEM. Dots represent individual values.
