# Supplementary figures and images for: P2X7 Receptor Upregulation in Huntington’s Disease Brains
Source: Front Mol Neurosci. 2020 Oct 6;13:567430. doi: 10.3389/fnmol.2020.567430 (PMC7573237; doi:10.3389/fnmol.2020.567430)

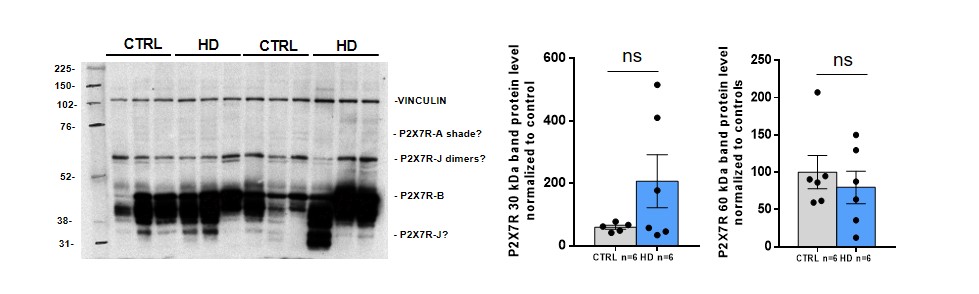

Supplement: FIGURE S1 — P2X7R protein levels detected with an N-terminus directed antibody in control and HD striatum. Blot image (left) is able to highlight multiple bands after prolonged exposure. The band at around 30 kDa could correspond to P2X7R-J. As shown by its quantification (right), such band does not significantly vary between HD and controls. Doublets can be observed at around 60 kDa, which could correspond to P2X7R-J dimers. However, no significant change occurs in HD samples. Only a light shade is observed in the range of P2X7R-A. Graphs show mean ± SEM. Dots represent individual values. Student’s t-test, ns: non-significant. [file Image_1.JPEG]

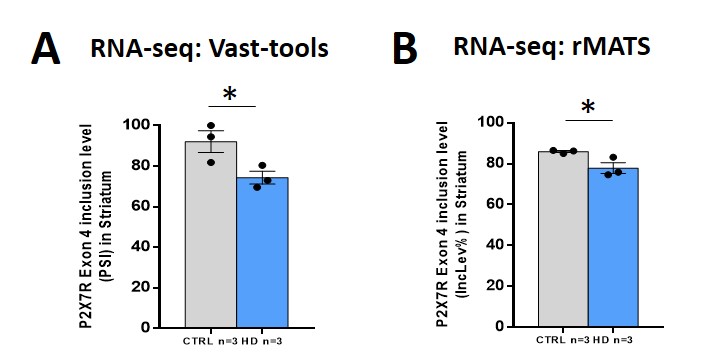

Supplement: FIGURE S2 — P2X7R exon 4 inclusion is decreased in HD. RNA-seq analysis of control and HD striatum by (A) Vast-tools result (Student’s t-test, ∗p < 0.05) was confirmed by (B) rMATS (∗FDR < 0.05). Graphs show mean ± SEM. Dots represent individual values. [file Image_2.JPEG]
